# Molecular and Cellular Pediatrics

**DOI:** 10.1186/s40348-014-0004-0

**Published:** 2014-09-04

**Authors:** Klaus-Michael Debatin

**Affiliations:** Klinik für Kinder- und Jugendmedizin, Universitätsklinikum Ulm, Eythstr. 24, Ulm, 89075 Germany

## Editorial

On behalf of the Editorial Board, I cordially welcome all physicians and scientists dedicated to pediatrics to contribute to the new journal *Molecular and Cellular Pediatrics.*

Although numerous high-ranking pediatric journals cover the field of clinical research, to date, scientific findings in cellular/molecular as well as translational research in pediatrics are mostly published in specialized journals of the respective area. In order to present a platform that covers all areas of basic and translational research related to pediatrics, the German Society of Pediatrics and Adolescent Medicine (Deutsche Gesellschaft für Kinder- und Jugendmedizin e.V., DGKJ) and Springer Verlag are happy to announce now the launching of this new international, open-access journal. I am confident that this journal will provide the appropriate forum for basic research-oriented pediatrics, a field that to date has remained largely neglected.

*Molecular and Cellular Pediatrics* is an international and interdisciplinary open-access journal publishing peer-reviewed articles on innovative research in pediatrics. All articles are made freely and permanently accessible online immediately upon publication.

The focus and the topics of this journal cover research areas relevant to pediatrics including rare diseases, genetic diseases, gene therapy, cardiology, endocrinology, gastroenterology, genetics, immunology, infectiology, nephrology, neurology, oncology and hematology, pneumology, metabolism, neonatology, and translational medicine in pediatrics.

*Molecular and Cellular Pediatrics* is dedicated to promoting the exchange and discussion of current molecular and cellular concepts in pediatric medicine. This includes frontline reviews of the latest developments, original research articles, and a forum for discussion of pathophysiological concepts.

*Molecular and Cellular Pediatrics* is an official journal of the German Society of Pediatrics and Adolescent Medicine (DGKJ). We thank Springer for making the launch of our journal possible. In addition, I especially thank our internationally recognized Editorial Board *(*http://www.molcellped.com*)*, whose members have volunteered to assist in this undertaking despite their many commitments. With their expertise covering all the abovementioned fields of research, a timely and competent review process is ensured. Thus, together with the professionals at Springer, we guarantee that your work is published with appreciation. I would like to encourage experienced as well as young colleagues to submit papers to *Molecular and Cellular Pediatrics*.

